# Impact of sports participation on gender inequality ideology among Chinese women—an empirical study based on the CGSS 2023 data

**DOI:** 10.3389/fpubh.2025.1721609

**Published:** 2025-12-18

**Authors:** Ping Fang, Peng yu Huo, Yan Lu, Shu sheng Shi, Lei Sun

**Affiliations:** 1 School of Sports Science, Nanjing Normal University, Nanjing, China; 2 Nanjing University of Science and Technology, Nanjing, China; 3 Nanjing University of Chinese Medicine, Nanjing, China

**Keywords:** China, gender inequality ideology, sports participation, women, empirical analysis

## Abstract

**Objectives:**

Influenced by traditional Chinese cultural values, Chinese women persistently face deeply rooted ideologies of gender inequality. Socioeconomic and demographic factors, along with social capital and engagement in sports activities, play a significant role in shaping this ideological framework. This study seeks to investigate the association between sports participation and women’s gender inequality ideology.

**Methods:**

Data were collected from the 2023 Chinese General Social Survey (CGSS). To explore the effect of sports participation on women’s gender inequality ideology, a multiple ordinary least squares (OLS) regression model was utilized. In addition, propensity score matching (PSM) was implemented to perform robustness checks, and an instrumental variables two-stage least squares (IV-2SLS) regression approach was also used to correct for possible bias in the analysis.

**Results:**

(1) The ideology underpinning gender inequality against women was more prominently manifested in the central and western regions. (2) Among individuals aged 15–44 years, those residing in non-agricultural households in eastern urban areas, with higher educational attainment, occupying social hierarchy levels 2–10, and exhibiting elevated socioeconomic status demonstrated a greater propensity to participate in women’s sports activities. (3) Compared to women who did not engage in sports activities, those who participated in sports exhibited a reduction of 4.069 in their endorsement of gender inequality ideology. This finding further substantiates the positive impact of sports participation on diminishing gender inequality beliefs among women. (4) After conducting endogenous and robustness analyses, the findings indicated a substantial level of robustness, and regular engagement in sports was shown to significantly reduce the average level of gender inequality ideology by 13.25, a magnitude notably greater than the estimates obtained from the OLS model.

**Conclusion:**

This study offers a comprehensive analysis of the changes in women’s perspectives on gender inequality, highlighting the pivotal role of sports participation in advancing gender equality among women in China. Future research efforts could enhance the understanding of the causal relationships between these variables by employing longitudinal tracking methodologies. It is advisable that forthcoming public policy initiatives prioritize the ongoing optimization of educational resources and the equitable allocation of fitness resources between urban and rural regions.

## Introduction

The ideology surrounding gender inequality serves as a key indicator of a country’s progress in addressing gender disparities and reflects the extent of traditional or unmodernized attitudes prevalent within its population. This ideology has played a pivotal role in the political, social, and economic empowerment of women worldwide, impacting the lives of countless individuals. Since the implementation of China’s reform and opening-up policies, rapid economic and social transformations have driven substantial changes in women’s gender ideology. This shift signifies a transition from the conventional paradigm of “men working outside the home and women managing domestic affairs” to a contemporary egalitarian discourse (1). However, when considering the practical implementation of gender equality ideologies, significant challenges persist. In China, women frequently encounter a “dual burden” in their daily lives. This term refers to the combination of professional obligations and primary domestic responsibilities, which is often viewed as a societal expectation. The present study hypothesizes that the disconnection between prevailing attitudes and actual behavior constitutes a central factor contributing to increased psychological and physical strain on women (2). It is also important to note that advancements in gender equality have been uneven, with significant disparities observed among different female demographics in China, particularly between urban and rural communities, across educational backgrounds, and along lines of socioeconomic status (3).

Gender role beliefs, sports participation, and sports media consumption are all significantly correlated with an individual’s gender typing in sports (4). The “Healthy China” strategy, a comprehensive initiative aimed at enhancing the overall health and well-being of the Chinese population, has played a pivotal role in this progress. In addition, the growing popularity of the concept of national fitness has contributed to increased sports participation among Chinese women. The “Healthy China 2030” blueprint identifies women as a key target group for the promotion of sports participation (5). The objective of this strategy is to advance the national health strategy through increased sports participation among women. Sports participation, as a routine practice integrating physical exercise, social interaction, and cultural meaning, provides a valuable theoretical lens for examining the micro-level formation of gender beliefs. However, existing research has predominantly focused on Western contexts or specific subgroups of female athletes (6, 7). Recent years have witnessed a notable surge in sports participation among Chinese women, which can be regarded as a manifestation of contemporary lifestyles (8). Analysis of available data indicates a positive trend in the participation rate of women in sports. It is important to note that sports participation is recognized not only for its physiological benefits but also as a social practice with the inherent potential to challenge and reshape gender norms (9, 10). From a theoretical perspective, the domain of sports has historically been a crucial site for the construction and performance of gender identities (11) and holds positive implications for women empowerment and the reconfiguration of gender norms (12). Although many studies have examined factors predicting individuals’ gender inequality ideology, little research has focused on the impact of sports participation on such ideologies. There is a lack of empirical investigations based on large-scale, representative samples of Chinese women. The relationship between sports participation and the formation of modern gender attitudes among Chinese women, along with its heterogeneous effects across age groups, household registration, marital status, region, educational background, and social hierarchy, requires systematic quantitative inquiry.

Accordingly, this study used 2023 data from the Chinese General Social Survey (CGSS) to systematically examine the impact of sports participation on gender inequality ideology among Chinese women. The present study, therefore, focused on rigorous econometric models to better identify the association between sports participation and gender inequality ideology, aiming to provide theoretical and practical references for improving women’s gender inequality ideology. The accelerated transformation of gender roles, coupled with the noteworthy economic and social transformations witnessed in contemporary China, provides a distinctive opportunity to delve into pivotal inquiries concerning sports participation and gender equality.

## Literature review

### Gender inequality ideology and sports participation

Gender inequality ideology in China over the past century began at an extreme, with the Confucianism of the Imperial era (13). During this period, the Wu Lun, the doctrine of filial piety, the Yin of being a woman, clan rules for marital relationships, and formal government laws all placed women in a very subservient position. Women’s educational, employment, and political opportunities were extremely limited. When multiple aspects of gender inequality are assembled together, it becomes evident that all societies exhibit both gender-egalitarian and gender-inegalitarian features (14). From women’s economic well-being and financial autonomy, through labor force participation and continuity of employment, to occupational attainment and economic rewards, the analysis confirms the existence of distinctive profiles of gender inequality (14). The theoretical underpinnings of both sociology and gender studies posit the notion that gender is not an innate, biologically determined construct; rather, it is a social and cultural construct (15). Individuals continuously accept and internalize a spectrum of gender ideologies, ranging from traditional to egalitarian, in socialization contexts such as family, school, and media (16). Liberal egalitarian gender ideology is widespread globally. Previous studies have indicated that egalitarian gender ideologies, which advocate for the equitable distribution of responsibilities between men and women in both household affairs and paid employment, have the potential to enhance the quality of life and promote greater happiness for all genders (17, 18). Gender inequality ideology is not merely a social injustice but a pervasive barrier to sustainable development, with detrimental consequences that resonate across economic, social, and individual domains (19, 20). Gender inequality ideology, being a stable social attitude, exerts a profound influence on individuals’ cognitive processes and behavioral decision-making.

It is evident that sports participation has the capacity to challenge traditional gender inequality ideologies. The active participation of women in sports that are traditionally regarded as “masculine” has been shown to have a number of beneficial effects. These include the enhancement of bodily autonomy and the cultivation of self-confidence. Furthermore, such participation can help develop a critical awareness of gender regulation (21, 22). Although many studies have examined factors predicting individuals’ gender ideology, little research has focused on the impact of sports participation on women’s gender inequality ideology. This study focuses on the relationship between sports participation and gender inequality ideology and proposes research hypotheses.

*Hypothesis 1*: Overall, sports participation will have a positive impact on women’s gender ideology.

### Factors influencing gender inequality ideology through sociodemographic variables

The strong ideological interaction between spouses presents a significant opportunity for exchanging egalitarian beliefs as society progresses toward more gender-equal norms (23). A total of five ideology profiles have been identified across all countries: egalitarian, egalitarian essentialism, intensive parenting, moderate traditional, and traditional (24). In addition, Shu XL et al. mapped gender ideologies globally by classifying individuals into four domains of ideological space: three varieties of egalitarianism—liberal egalitarian, egalitarian essentialist, and flexible traditionalist—and one traditional ideology, traditional essentialist (25). Economic development is associated with liberal egalitarian and egalitarian essentialist attitudes, both supporting gender inequality ideology reforms. Ideologies mixing gender essentialist and egalitarian views appear to have replaced traditional inequality ideologies, even in countries offering some institutional support for gendered separate spheres. Still, a significant minority of couples challenge dominant norms, particularly in their paid work arrangements. Bornatici et al. advocated for policy reforms that establish supportive institutions, enabling couples to align their behaviors with their gender ideologies and paving the way for greater equality in the future (26).

The concept of gender ideology is understood to be in a constant state of evolution, being continuously shaped and reshaped through a dynamic interplay with social structures, including, but not limited to, educational systems, labor markets, and family institutions (27–29). Supporting this, this study indicates that a higher educational background is a robust predictor of stronger gender equality consciousness among women, as education directly empowers them to challenge traditional gender inequality roles (30). Respondents with a lower level of education and men have higher chances of holding non-egalitarian ideologies. Longitudinal studies suggest that women who maintain continuous employment are most likely to hold egalitarian gender attitudes. Furthermore, women who return to the workforce after childbirth may develop even more gender equitable attitudes (31), yet the parallel shift in expecting men to share domestic responsibilities has not occurred. This discrepancy creates a paradoxical and strained contemporary gender inequality ideology. The findings demonstrated that age, education level, country of education, and wife’s employment were not significant predictors of egalitarian ideology after controlling for acculturation level. However, when all predictor variables were considered together, 33% of the variance in gender role ideology could be explained—higher than any single variable could predict alone (32). To explore the influence of various social demographic factors on gender inequality ideology, a key research question arises: Does the impact of sports participation on women’s gender inequality ideology vary by age, household registration, marital status, region, educational background, social hierarchy, and socioeconomic status? Based on this question, the second research hypothesis was proposed.

*Hypothesis 2*: The impact of sports participation on women’s gender ideology differs according to age, household registration, marital status, region, educational background, social hierarchy, and socioeconomic status.

## Methods

### Measure of variables

#### Data sources

All data used in this study were obtained from the 2023 CGSS. Comprehensive data collection was carried out across multiple levels, including society, communities, households, and individuals. This approach summarizes trends in social change, explores significant scientific and practical issues, promotes openness and sharing in domestic scientific research, provides data resources for international comparative studies, and serves as a multidisciplinary platform for economic and social data collection. According to the 2023 CGSS dataset, the data used in this study cover 31 provincial-level cities in China, comprising a total of 11,326 observations, ensuring its representativeness. In this study, the data were organized after removing missing variables, non-responses, and samples that did not conform to objective logic, resulting in 2,128 valid observations.

#### Sports participation

The variable representing sports participation was operationalized as a dichotomous measure, defined as “participation in sports within a week.” The original survey item asked the following question: “Over the past year, have you frequently engaged in physical exercise during your free time?” Responses indicating “never” were coded as 0, signifying no involvement in sporting activities. In contrast, all other responses were coded as 1, indicating engagement in sports.

#### Gender inequality ideology

The dependent variable in this study was the ideology of gender inequality. Gender inequality ideology was primarily measured using the scale from the CGSS questionnaire. Specifically, it included four items: “Men put their careers first, women put their families first,” “Men are naturally more capable than women,” “Better to marry well than to do well,” and “When the economy is sluggish, women employees should be laid off first.” The response options for each item were “Disagree,” “Relatively disagree,” “It does not matter whether you agree or disagree,” “relatively agree,” and “completely agree.” Considering the differences in project content and measurement standards, factor analysis was used to extract common factors. In Table 1, the results of the factor analysis show that the reliability coefficient of the gender inequality ideology scale reached 0.727, indicating that its internal consistency is at an acceptable level. The KMO value was 0.744, meeting the requirements of statistical analysis (Table 1). By standardizing the “gender inequality ideology factor,” a gender inequality ideology variable with a score range from 0 to 100 was generated. The higher the score, the more unequal and traditional the gender ideology. Conversely, the lower the score, the more modern and egalitarian it tends to be. This standardization was performed by subtracting the minimum factor value from each original factor value, dividing the result by the range of factor values, and finally multiplying by 100.

**Table 1:** Factor analysis results of gender inequality ideology.

Item	Mean	SD	Gender inequality ideology factor loading
Men put their careers first, women put their families first	3.108	1.426	0.7865
Men are naturally more capable than women	2.789	1.379	0.8040
Better to marry well than to do well	3.085	1.373	0.7289
When the economy is sluggish, women employees should be laid off first	1.882	1.108	0.6419
Root of characteristic	2.201		
KMO	0.744		

#### Control variables

Based on the research findings of the existing literature and the availability of variables in the CGSS data, socioeconomic and demographic factors were included, encompassing age, household registration, marital status, region, educational background, social hierarchy, and socioeconomic status (Table 2). (1) Age was divided into three categories: Young (15–44 years old) = 1, middle-aged (45–59 years old) = 2, and older (60 years old and above) = 3. (2) Household registration was measured based on a question asking the respondents about current household registration status and coded as 0 for “agricultural household registration” and 1 for “non-agricultural household registration.” (3) Marital status was coded as a dichotomous variable, with 0 indicating married and 1 indicating unmarried. (4) Region included three categories: East, middle, and west. (5) Educational background was divided into 13 levels. (6) Social hierarchy had 10 options, ranging from 1 point to 10 points. (7) Socioeconomic status included five categories: Lower stratum, lower-middle stratum, middle stratum, upper-middle stratum, and upper stratum.

**Table 2:** Variables definition.

Type	Variable	Code	Variable declaration
Dependent variable	Gender in quality ideology	a42	Gender in quality ideology score
Independent variable	Sports participation	a3009	Participation = 1; Not participating = 0
Control variables	Age	a3a	Young (15–44 years old) = 1; Middle-aged (45–59 years old) = 2; Older adults (60 years old and above) = 3
	Household registration	a18	Non-agricultural household = 1; Agricultural household registration = 0
	Marriage	a69	Married = 1; Unmarried = 0
	Region	s41	East = 1; Middle = 2; West = 3
	Educational background	a72	No education = 1; Private school and literacy class = 2; Primary school = 3; Junior high school = 4; Vocational high school = 5; Regular high school = 6; Technical secondary school = 7; Technical school = 8; College (adult higher education) = 9; College (formal higher education) = 10; Undergraduate (adult higher education) = 11; Bachelor degree (formal higher education) = 12; Graduate student or above = 13
	Social hierarchy	a43a	1 point = 1; 2 points = 2; 3 points = 3; 4 points = 4; 5 points = 5; 6 points = 6; 7 points = 7; 8 points = 8; 9 points = 9; 10 points = 10
	Socioeconomic status	a43e	Lower stratum = 1; Lower-middle stratum = 2; Middle stratum = 3; Upper-middle stratum = 4; Upper stratum = 5

#### Analytic strategy

The present study employed a multiple linear regression model to investigate the impact of female participation in sports on changes in gender inequality ideology. The model is expressed as follows:


$$\begin{array}{c} \text{Gender inequality ideology}\;i=\beta 0+\beta 1\;\text{Sports participation}\;i\\ +\sum_{i=2}^{n}\mathrm{\beta i}Xi+\mathrm{\varepsilon i} \end{array}$$

Propensity score matching (PSM) was adopted to perform robustness checks. In addition, an instrumental variables two-stage least squares (IV-2SLS) approach was used for constructing the concept of sport participation to deal with endogeneity.

## Results

### Descriptive statistics

As illustrated in Table 3, the descriptive statistical analysis revealed that 54.320% of the participants were women engaged in sports activities, whereas 45.680% of the women did not partake in physical exercise. In addition, a relatively high proportion of women who were married, middle-aged or older, and registered as agricultural household members was observed. Concerning the regional distribution of the study sample, the number of participants from the central, western, and eastern regions was observed to be approximately equivalent. The majority of the sample had teaching backgrounds in primary schools, junior high schools, and regular high schools, comprising 71.140% of the total sample. Regarding social hierarchy, the majority of the participants were concentrated in the 1–6 point range, with a significant proportion in the 5-point category. Only a small number of individuals attained high scores. In terms of socioeconomic status, the majority of the participants were concentrated in the middle group, with only 0.610% of the sample falling into the upper stratum.

**Table 3:** Descriptive statistics of variables.

Variable	Classification	Freq	%	*Cum*	Mean	SD	Min	Max
Sports participation	Not participating	972	45.680	45.680	0.543	0.498	0	1
	Participation	1,156	54.320	100.000				
Age	Young (15–44 years old)	514	24.150	24.150	2.128	0.789	1	3
	Middle-aged (45–59 years old)	742	34.870	59.020				
	Older adults (60 years old and above)	872	40.980	100.000				
Household registration	Agricultural household registration	1,525	71.660	71.660	0.283	0.451	0	1
	Non-agricultural household	603	28.340	100.000				
Marriage	Unmarried	67	3.150	3.150	0.969	0.175	0	1
	Married	2061	96.850	100.000				
Region	East	722	33.930	33.930	1.996	0.822	1	3
	Middle	692	32.520	66.450				
	West	714	33.550	100.000				
Educational background	No education	148	6.950	6.950	4.996	2.783	1	13
	Private school and literacy class	9	0.420	7.380				
	Primary school	484	22.740	30.120				
	Junior high school	750	35.240	65.370				
	Vocational high school	22	1.030	66.400				
	Regular high school	280	13.160	79.560				
	Technical secondary school	91	4.280	83.830				
	Technical school	16	0.750	84.590				
	College (adult higher education)	85	3.990	88.580				
	College (formal higher education)	86	4.040	92.620				
	Undergraduate (adult higher education)	59	2.770	95.390				
	Bachelor degree (formal higher education)	90	4.230	99.620				
	Graduate student or above	8	0.380	100.000				
Social hierarchy	1 point	211	9.920	9.920	4.411	1.868	1	10
	2 points	148	6.950	16.870				
	3 points	252	11.840	28.710				
	4 points	294	13.820	42.530				
	5 points	767	36.040	78.570				
	6 points	256	12.030	90.600				
	7 points	97	4.560	95.160				
	8 points	60	2.820	97.980				
	9 points	12	0.560	98.540				
	10 points	31	1.460	100.000				
Socioeconomic status	Lower stratum	410	19.270	19.270	2.369	0.868	1	5
	Lower-middle stratum	653	30.690	49.950				
	Middle stratum	947	44.500	94.450				
	Upper-middle stratum	105	4.930	99.390				
	Upper stratum	13	0.610	100.00				

### Gender inequality ideology and sports participation across different socioeconomic and demographic groups

Generally, the proportion of middle-aged and older women with agricultural household registration who held gender inequality ideology was significantly higher than that of the residents with gender equality ideology (29.371%), and the average social justice perception among the residents was 2.128. Moreover, the ideology of gender inequality was more pronounced among the women in the central and western regions, with more than 50% of these women lacking education, having not attended private school or literacy classes, and having not completed primary school, exhibiting a strong adherence to gender inequality ideology. The present study revealed that the women who scored 1, 9, and 10 on the social hierarchy scale exhibited higher levels of gender inequality ideology, with a proportion exceeding 50%. However, the women with lower economic status exhibited higher levels of gender inequality ideology compared to the women in other economic strata. In terms of sports participation, the women who were young (15–44 years old), had non-agricultural household registration, lived in eastern cities, had a higher educational background, scored 2–10 on the social hierarchy scale, and belonged to higher socioeconomic strata showed higher levels of sports participation initiative (Table 4).

**Table 4:** Gender inequality ideology and sports participation among different demographic groups.

Variable	Classification	Gender ideology	Sports participation(%)
Age	Young (15–44 years old)	29.371***	64.786***
	Middle-aged (45–59 years old)	42.748***	55.660***
	Older adults (60 years old and above)	50.294***	47.018***
Household registration	Agricultural household registration	45.954***	46.951***
	Non-agricultural household	34.148***	73.768***
Marriage	Unmarried	45.005	58.209
	Married	42.531	54.197
Region	East	38.630***	62.188***
	Middle	45.813***	52.457***
	West	43.527***	48.179***
Educational background	No education	54.305***	23.649***
	Private school and literacy class	61.605***	22.222***
	Primary school	53.272***	40.289***
	Junior high school	43.459***	52.400***
	Vocational high school	36.726***	72.727***
	Regular high school	36.696***	63.214***
	Technical secondary school	34.357***	60.440***
	Technical school	32.080***	75.000***
	College (adult higher education)	31.642***	76.471***
	College (formal higher education)	24.724***	81.395***
	Undergraduate (adult higher education)	26.150***	86.440***
	Bachelor degree (formal higher education)	27.529***	85.556***
	Graduate student or above	17.866***	100.000***
Social hierarchy	1 point	51.191***	37.915***
	2 points	45.647***	47.297***
	3 points	45.218***	46.825***
	4 points	41.061***	51.020***
	5 points	41.467***	56.975***
	6 points	38.748***	66.406***
	7 points	34.561***	65.979***
	8 points	38.793***	70.000***
	9 points	50.599***	66.667***
	10 points	52.767***	54.839***
Socioeconomic status	Lower stratum	49.894***	39.268***
	Lower-middle stratum	40.407***	54.977***
	Middle stratum	40.925***	59.556***
	Upper-middle stratum	43.648***	60.952***
	Upper stratum	37.704***	61.538***

Statistical significance was assessed by one-way ANOVA followed by Tukey’s post-hoc test; ***, **, * represent significant at the 1, 5, and 10% levels, respectively, which refer to the (8).

### The influence of sports participation on women’s gender inequality ideology

The establishment of the multiple linear regression model included gender ideology as the explanatory variable, sports participation as another explanatory variable, and socioeconomic and demographic characteristics and social capital as control variables (Table 5). The first model selected only core explanatory variables, whereas the second model included control variables and core explanatory variables on the basis of the first model. The study concluded that sports participation exerts a significant positive influence on women’s gender inequality ideology, thereby substantiating Hypothesis 1. Model 1 established a strong, raw negative relationship between sports participation and gender inequality ideology. This suggested a robust baseline connection. The findings of Model 2 demonstrated that sports participation continues to exert a substantial positive influence on women’s gender inequality ideology, even after accounting for key control variables, including socioeconomic and demographic characteristics and social capital. Compared to the residents who had not participated in sports in the previous year, the gender inequality ideology of those who had participated decreased by 4.069. This indicates that a substantial portion of the initial effect can be explained by the demographic and socioeconomic characteristics of individuals who participate in sports. However, a significant independent effect of sports participation remains.

**Table 5:** OLS estimation results of the impact of sports participation on gender inequality ideology.

Variables	Model 1	Model 2
Sports participation (not participating = 0)	−10.971_***_ (1.051)	−4.069*** (1.042)
Age [young (15–44 years old)]		
Middle-aged (45–59 years old)		9.998*** (1.308)
Older adults (60 years old and above)		17.201*** (1.350)
Non-Agricultural household registration (agricultural household registration = 0)		−7.195*** (1.172)
Married (unmarried = 0)		−0.160 (2.589)
Region (East = 1)		
Middle		3.344** (1.215)
West		−0.582 (1.236)
Educational background (no education = 1)		
Private school and literacy class		4.831 (6.691)
Primary school		2.518 (2.055)
Junior high school		−3.152 (2.018)
Vocational high school		−4.142 (4.644)
Regular high school		−8.834*** (2.226)
Technical secondary school		−8.414** (2.979)
Technical school		−11.537** (4.108)
College (adult higher education)		−9.650*** (2.836)
College (formal higher education)		−13.070*** (2.954)
Undergraduate (adult higher education)		−10.960*** (3.314)
Bachelor degree (formal higher education)		−9.465*** (2.834)
Graduate student or above		−12.627 (6.933)
Social hierarchy (1 point = 1)		
2 points		−3.390 (2.491)
3 points		−3.505 (2.133)
4 points		−5.059* (2.087)
5 points		−4.478* (1.897)
6 points		−5.046* (2.260)
7 points		−7.725** (2.910)
8 points		−5.470 (3.383)
9 points		3.107 (4.802)
10 points		3.953 (5.454)
Socioeconomic status (lower stratum = 1)		
Lower-middle stratum		−4.145** (1.454)
Middle stratum		−1.842 (1.547)
Upper-middle stratum		−0.335 (2.694)
Upper stratum		−13.808 (7.067)
_cons	48.569*** (0.805)	45.617*** (3.793)
*N*	2,128	2,128
*R* ^2^	0.050	0.234
*F*	109.056	23.181

*p < 0.05; **p < 0.01; ***p < 0.001.

### Results of the PSM robustness test

The results of the classical ordinary least squares (OLS) regression model provided an inconclusive answer to the question regarding the relationship between sports participation and the ideology of gender inequality among women. It is evident that participation in sporting activities is characterized by a discernible element of selective bias. To control the influence of selection bias caused by confounding variables, this study mainly selected the methods of nearest neighbor matching and kernel matching for tendency score matching analysis. After matching using the propensity score matching method, all variables passed the balance test. Based on this, we estimated the real impact of sports participation on women’s gender inequality ideology using the method of propensity score matching. The results of the PSM estimation of the impact of sports participation on gender inequality ideology, which is shown that the two matching methods results are significant at the 1% statistical level. And the average ATT of nearest neighbor matching and kernel matching is −3.939, indicating that women’s participation in sports has reduced gender inequality ideology. Therefore, after correcting for selective bias, the propensity score matching method further confirmed the positive influence of sports participation on the modernization of women’s gender inequality ideology.

### Endogenous treatment

To address the endogenous problem, the IV-2SLS method was employed using instrumental variables. Table 6 reports the estimation results of the instrumental variable regression model. The results showed that all three models passed the weak instrumental variable test and Durbin–Wu–Hausman test and verified that “watching sports games on the spot” and “going to a gymnasium or gym for exercise” are effective instrumental variables, which can reduce the interference caused by the endogenous bias of the model. The estimation results of the instrumental variable model further confirmed that the inhibitory effect of sports participation on gender inequality ideology is robust and reliable. In the instrumental variable model, sports participation still showed a positive effect on gender inequality ideology, further proving that physical exercise has a robust inhibitory effect on gender inequality ideology. Specifically, in the two instrumental variables models, regular participation in sports reduced the average gender inequality ideology by 13.25 points, which was significantly higher than the estimated results of the OLS model.

**Table 6:** Results of the IV-2SLS.

Categories	(1)	(2)	(3)
	IV-2SLS	IV-2SLS	IV-2SLS
Sports participation (not participating = 0)	−11.53*** (3.969)	−16.31*** (5.058)	−13.25*** (3.469)
Age [young (15–44 years old = 1)]			
Middle-aged (45–59 years old)	9.843*** (1.328)	9.743*** (1.362)	9.807*** (1.337)
Older adults (60 years old and above)	16.57*** (1.419)	16.16*** (1.467)	16.42*** (1.416)
Non-Agricultural household registration (agricultural household registration = 0)	−6.009*** (1.316)	−5.250*** (1.442)	−5.737*** (1.291)
Married (unmarried = 0)	−0.718 (2.611)	−1.075 (2.670)	−0.847 (2.621)
Region (East = 1)			
Middle	3.326*** (1.220)	3.315*** (1.244)	3.322*** (1.227)
West	−0.744 (1.238)	−0.848 (1.258)	−0.781 (1.242)
Educational background (no education = 1)			
Private school and literacy class	4.853 (6.583)	4.866 (6.619)	4.858 (6.590)
Primary school	3.641* (2.117)	4.360** (2.219)	3.900* (2.115)
Junior high school	−1.448 (2.169)	−0.358 (2.345)	−1.057 (2.147)
Vocational high school	−0.944 (4.902)	1.103 (5.182)	−0.209 (4.866)
Regular high school	−6.549*** (2.519)	−5.086* (2.758)	−6.023** (2.473)
Technical secondary school	−6.464** (3.172)	−5.216 (3.372)	−6.016* (3.167)
Technical school	−8.417* (4.431)	−6.420 (4.852)	−7.700* (4.437)
College (adult higher education)	−6.709** (3.226)	−4.827 (3.516)	−6.033* (3.158)
College (formal higher education)	−9.896*** (3.336)	−7.864** (3.724)	−9.166*** (3.288)
Undergraduate (adult higher education)	−7.455** (3.725)	−5.211 (4.213)	−6.649* (3.698)
Bachelor degree (formal higher education)	−6.216* (3.228)	−4.136 (3.568)	−5.469* (3.143)
Graduate student or above	−8.886 (7.050)	−6.492 (7.274)	−8.026 (7.003)
Social hierarchy (1 point = 1)			
2 points	−2.815 (2.524)	−2.447 (2.572)	−2.683 (2.531)
3 points	−3.142 (2.151)	−2.910 (2.185)	−3.059 (2.157)
4 points	−4.733** (2.098)	−4.525** (2.135)	−4.658** (2.105)
5 points	−3.829** (1.936)	−3.413* (1.985)	−3.679* (1.937)
6 points	−4.084* (2.320)	−3.468 (2.370)	−3.863* (2.309)
7 points	−6.793** (2.970)	−6.197** (3.057)	−6.579** (2.978)
8 points	−4.010 (3.547)	−3.076 (3.693)	−3.674 (3.555)
9 points	4.451 (4.462)	5.311 (4.337)	4.760 (4.379)
10 points	4.844 (5.517)	5.414 (5.627)	5.049 (5.543)
Socioeconomic status (lower stratum = 1)			
Lower-middle stratum	−3.523** (1.499)	−3.125** (1.571)	−3.380** (1.507)
Middle stratum	−1.261 (1.608)	−0.888 (1.659)	−1.127 (1.611)
Upper-middle stratum	0.198 (2.730)	0.540 (2.767)	0.321 (2.735)
Upper stratum	−12.96* (7.074)	−12.42* (7.151)	−12.76* (7.091)
_cons	47.39*** (3.910)	48.53*** (4.030)	47.80*** (3.895)
Weak Instrumental Variable Test	231.542***	122.942***	155.23***
Durbin–Wu–Hausman Test	3.871*	6.56234*	7.891**
*N*	2,128	2,128	2,128
r2_a	0.202	0.168	0.192

*p < 0.05; **p < 0.01; ***p < 0.001.

## Discussion

The feminist approach centralized adolescent girls’ voices, thereby recognizing that physical activity is rooted in patriarchal constructions that position girls as naturally uninterested in sports and activity. Gender-focused interventions can actively address these stereotypes by listening to girls (33). The function of sex stereotypes and gender roles in the sex differences observed in sports and exercise has been extensively investigated in sport psychology, with previous studies showing that stereotypes are internalized into the self during the socialization process (34). The data analysis revealed that the ideology of gender inequality was more prominently observed among the women in the young and middle-aged cohorts. From an age-related perspective, young and middle-aged women who engage in sports are more inclined to uphold egalitarian gender ideologies. Women in early and middle adulthood occupy a critical life stage characterized by the navigation of romantic relationships, career advancement, and the allocation of familial responsibilities. Among the women without agricultural household registration, those residing in urban areas who engaged in sports activities exhibited more progressive gender ideologies. The abundance of sports resources and facilities in urban areas has been identified as a key factor facilitating women’s participation in various sports projects and the development of consistent sporting habits. Women in the eastern region, characterized by a more advanced economy, have been significantly exposed to concepts of gender equality via media, educational opportunities, and international interactions. Consequently, the marginal effect of sports participation has been relatively small. Traditional gender inequality ideologies still exist in the central region, but rapid socioeconomic and cultural development has made sports an effective means of promoting gender equality. Women with higher educational attainment tend to endorse gender equality principles and demonstrate increased participation in physical exercise. The findings related to social hierarchy and socioeconomic status indicate that women belonging to the upper-middle socioeconomic strata are more likely to engage in sports activities and show reduced adherence to gender inequality ideologies. Similarly, higher socioeconomic status is associated with increased participation in physical exercise and lower endorsement of gender inequality beliefs. Survey data indicate that married women, those with business education, and those in the top levels of their organizations are more likely to address gender inequality (35). Conventional gender ideologies exert the most immediate pressure and generate significant contradictions during this phase. When women establish families and advance from ordinary employees to senior positions through business training, they gain the absolute authority to challenge societal notions of gender inequality. For those living in areas with lower levels of gender inequality, the daughter is a stronger predictor of egalitarian gender ideology (36). Consequently, the ideology surrounding gender inequality affecting women is also shaped by social and environmental factors.

In the present study, a regression model was used to analyze the relationship between sports participation and gender inequality ideology. The findings indicated that sports participation could serve as a mitigating factor in the expression of women’s gender inequality ideology, even after controlling for variables. The findings of the present study indicated that, compared to agricultural household registration, non-agricultural household registration exerted a considerable negative influence on women’s gender inequality ideology. Agricultural economies often rely on a family-labor model with clearly defined gender roles (e.g., men performing fieldwork and women performing domestic duties). Non-agricultural economies offer a wider variety of jobs for all genders, weakening the material basis for traditional ideologies. Non-agricultural household registration is typically associated with centers of education, diverse media, and progressive social movements, exposing residents to more egalitarian ideas. The middle-aged and older women exhibited a positive association with gender inequality ideology, suggesting that women in these age groups in the central region strongly endorse beliefs aligned with gender inequality. Non-agricultural household registration, higher educational background, middle social hierarchy, and lower-middle socioeconomic status are negatively correlated with gender inequality ideology. The experience of women in hypogamy is characterized by suboptimal physical and mental health, a phenomenon that can be attributed to the conflict between this social group and traditional gender ideologies surrounding marriage. This conflict is a significant contributing factor to the stress experienced by women in hypogamy (37). However, the recent increase in women’s education and the subsequent change in attitudes toward assortative marriages may alter this trend (37). Higher educational attainment among women provides advantages in the workplace. The effect is nuanced but clear. Higher levels of formal education are consistently and strongly associated with lower gender inequality ideology, compared to having no education. A positive gender ideology is associated with being female, younger age, urban living, higher income, and higher levels of maternal and paternal education (38). In addition, women’s exercise habits are influenced by traditional gender ideologies, and women have long been excluded and treated unfairly in the realm of exercise. The individuals who were young, lived in a city (i.e., an urban area), had a full-time job, reported good physical health, and had enough income to meet their daily needs were more likely to participate in sports regularly. In addition, higher education attainment has been shown to be associated with greater cultural capital, which enables the translation of the experience of equality in sports into ideologies and practices of equality in broader social life. Moreover, the findings were non-linear but suggested that individuals who place themselves in the middle of the social hierarchy (4–7 points) hold significantly less gender-inegalitarian views than those at the bottom (1 point). According to theories such as the “Resource Hypothesis,” individuals with greater economic security and resources have less need to adhere to rigid hierarchical social orders to maintain a sense of stability. They can afford to be more egalitarian. Being in the lower-middle stratum significantly reduces inegalitarian views compared to the lower stratum. Individuals at the very bottom of the social ladder might be in direct competition for scarce resources, potentially fostering more traditional and authoritarian worldviews as a coping mechanism.

To verify the causal relationship between sports participation and gender inequality ideology, the 2SLS method using instrumental variables was employed. The analysis demonstrated that sports participation exerts a significant effect on such inequality ideology. Participation in sports has been demonstrated to have a positive impact on the development of confidence, the cultivation of a competitive spirit, and the enhancement of collaborative abilities. Moreover, it has been observed to influence workplace competition, major family decisions, and the negotiation of marital relationships. The immediate positive feedback that results from sports participation can also contribute to the promotion of positive gender ideologies. Since sports participation has an independent and significant effect, public policy should actively fund and promote sports programs in schools and communities, with a specific focus on making them accessible and encouraging for female individuals. This is not just about health but also about fostering a more egalitarian society. The strong age effect highlights the need for targeted awareness campaigns for older populations. Public service announcements, community workshops, and storylines in popular media aimed at older demographics can help modernize attitudes. The stark divide based on Hukou status highlights the need to integrate gender equality education into rural revitalization strategies. This includes promoting women’s non-agricultural entrepreneurship, challenging traditional land inheritance practices, and improving access to information in rural areas. The powerful effect of education, particularly formal pathways, underscores that investing in quality education is a long-term investment in gender equality. Curricula should explicitly include gender studies and foster critical thinking about social norms from an early age. The effects of social hierarchy and socioeconomic status suggest that policies aimed at reducing overall economic inequality and providing social safety nets may indirectly promote more progressive gender attitudes by reducing the existential anxieties that can fuel traditionalism. In conclusion, the data show that gender ideology is not a fixed trait but is malleable and shaped by specific life experiences—sports, education, urban living, and economic security. Therefore, practical interventions should target these key socializing institutions and structural factors.

## Conclusion and recommendations

The findings confirm the positive role of sports participation in fostering egalitarian gender ideology. The aforementioned conclusions underscore the practical significance of engaging in sports for the promotion of gender equality and the establishment of a healthy China. It is crucial to recognize the pivotal role of sports participation in shaping social gender ideology and to foster a conducive environment for the promotion of women’s sports participation. In addition, the evidence suggests that sports participation may also function as a significant mechanism reinforcing traditional gender inequality ideologies, especially among female participants.

The 2023 National Fitness Activity Survey Report indicates that the proportion of women classified as “regular exercisers” has reached 35.9%, reflecting a significant and rapid increase in female participation in sports. In this context, it is crucial to acknowledge the role of sports engagement in fostering progressive gender attitudes and to cultivate a more supportive social environment that encourages women’s involvement in athletic activities. To this end, there is a need to strengthen public awareness initiatives through digital media platforms, community-based workshops, and other outreach channels. These efforts aim to disseminate knowledge related to sports, improve physical skills, and guide women in developing healthy attitudes toward both sports participation and gender roles, thereby increasing their motivation to engage in regular exercise. Moreover, the implementation of policy interventions, such as “sports consumption vouchers” or “fitness reward programs,” specifically designed for women, particularly those in the appropriate age groups in central and western regions, could effectively lower participation barriers and promote the development of sustainable exercise habits.

To enhance the impact of sports participation on gender attitudes, it is imperative to strategically reallocate public sports resources and tailor service content to the specific needs of women. Empirical findings indicate that young women with non-agricultural household registration in eastern China experience the most significant gains in egalitarian gender attitudes following sports participation. Therefore, policy efforts should prioritize the continuous optimization of the urban–rural distribution of fitness resources, including upgrading public sports infrastructure and directing allocations toward underserved western and rural regions. Furthermore, a nuanced approach is required to address the diverse demands of different women subgroups. Communities and enterprises should be encouraged to design and implement targeted programs. Examples include “Community Parent–Child Sports Parks,” which reconcile familial responsibilities with personal health needs and are designed for middle-aged and older women, and “Rural Fun Fitness Exercises,” which are aimed at boosting participation among women in rural areas.

### Limitations

This study has several limitations. First, the scope of the CGSS questionnaire data precluded a more granular causal analysis of the relationship between sports participation and women’s gender ideology, particularly with respect to specific dimensions such as exercise frequency and type. Second, constraints inherent in the survey content hindered the investigation of the mediating mechanisms through which sports participation affects gender inequality ideology. Furthermore, although self-efficacy is a significant determinant of gender inequality ideology, relevant data were not available in the current CGSS dataset. Addressing these limitations in future research will require conducting follow-up surveys or specialized studies explicitly designed to explore these aspects.

## Data Availability

Publicly available datasets were analyzed in this study. This data can be found here: http://cgss.ruc.edu.cn/.
